# Respiratory Syncytial Virus Inhibits Ciliagenesis in Differentiated Normal Human Bronchial Epithelial Cells: Effectiveness of N-Acetylcysteine

**DOI:** 10.1371/journal.pone.0048037

**Published:** 2012-10-31

**Authors:** Manuel Mata, Irene Sarrion, Miguel Armengot, Carmen Carda, Isidoro Martinez, Jose A. Melero, Julio Cortijo

**Affiliations:** 1 Research Foundation of the University General Hospital of Valencia, Valencia, Spain; 2 Centro de Investigación Biomédica en Red (CIBER) de Enfermedades Respiratorias, Valencia, Spain; 3 University of Valencia, Valencia, Spain; 4 University General Hospital of Valencia, Valencia, Spain; 5 Unidad de Interacción Virus-Célula, Centro Nacional de Microbiología, Instituto de Salud Carlos II, Madrid, Spain; 6 Unidad de Biología Viral, Centro Nacional de Microbiología, Instituto de Salud Carlos II, Madrid, Spain; National Jewish Health, United States of America

## Abstract

Persistent respiratory syncytial virus (RSV) infections have been associated with the exacerbation of chronic inflammatory diseases, including chronic obstructive pulmonary disease (COPD). This virus infects the respiratory epithelium, leading to chronic inflammation, and induces the release of mucins and the loss of cilia activity, two factors that determine mucus clearance and the increase in sputum volume. These alterations involve reactive oxygen species-dependent mechanisms. The antioxidant N-acetylcysteine (NAC) has proven useful in the management of COPD, reducing symptoms, exacerbations, and accelerated lung function decline. NAC inhibits RSV infection and mucin release in human A549 cells. The main objective of this study was to analyze the effects of NAC in modulating ciliary activity, ciliagenesis, and metaplasia in primary normal human bronchial epithelial cell (NHBEC) cultures infected with RSV. Our results indicated that RSV induced ultrastructural abnormalities in axonemal basal bodies and decreased the expression of β-tubulin as well as two genes involved in ciliagenesis, *FOXJ1* and *DNAI2*. These alterations led to a decrease in ciliary activity. Furthermore, RSV induced metaplastic changes to the epithelium and increased the number of goblet cells and the expression of *MUC5AC* and *GOB5*. NAC restored the normal functions of the epithelium, inhibiting ICAM1 expression, subsequent RSV infection through mechanisms involving nuclear receptor factor 2, and the expression of heme oxygenase 1, which correlated with the restoration of the antioxidant capacity, the intracellular H_2_O_2_ levels and glutathione content of NHBECs. The results presented in this study support the therapeutic use of NAC for the management of chronic respiratory diseases, including COPD.

## Introduction

Human respiratory syncytial virus (RSV; genus *Pneumovirus*, family *Paramixoviridae*) is an important pathogen that causes serious infection in people of all ages, including children, healthy and sick adults, and elderly individuals [1], [2]. This virus results in persistent infection, leading to chronic inflammation through mechanisms involving continuous stimulation of the immune system [3], [4], [5]. Persistent RSV infection appears to occur in certain individuals with chronic obstructive pulmonary disease (COPD), where RSV has been associated with exacerbation of the disease, the main cause of morbidity in patients with COPD [6], [7].

During COPD exacerbations, an increase occurs in sputum volume, which in the airways is the result of a balance between the ciliary beat of epithelial cells and mucin production [8]. Both processes are affected by RSV, which induces the destruction of ciliated epithelial cells and the expression of *MUC5AC*, the predominant mucin gene expressed in human airways [9], [10], [11], [12].

The involvement of reactive oxygen intermediates (ROIs) as mediators of the epithelial cell damage seen during exacerbations seems clear [13]. The source of these oxidants may be leukocytes via xanthine oxidase, which is increased in influenza-infected lungs, or lung epithelial cells themselves [13], [14], [15]. ROIs are necessary for RSV infection and are involved in the inflammatory response of host cells [16].

N-acetylcysteine (NAC) is a thiol compound that acts directly as a free radical scavenger and as a precursor of reduced glutathione (GSH) [17]. This molecule reduces the number and impact of COPD exacerbations [18] and also the inflammatory response in epithelial cells infected with respiratory viruses [19]. Recently, the effectiveness of NAC for inhibiting *MUC5AC* induction in A549 cells infected with RSV has been reported [16]. Although the anti-mucolitic effects of NAC are well established, little is known about its effects on ciliagenesis in human airway epithelial cells.

The main objective of this study was to analyze the effects of NAC in an *in vitro* model of RSV infection developed on air–liquid interface (ALI)-differentiated normal human bronchial epithelial cells (NHBECs). We studied the effects of this drug on viral replication, ciliary activity, ciliagenesis, and mucin production as well as its antioxidant effects by measuring the total antioxidant status (TAS), the intracellular H_2_O_2_ and glutathione levels and the expression of nuclear receptor factor 2 (Nrf2), heme oxygenase 1 (HO1), and ICAM1.

## Results

### Effect of NAC on virus replication

**Figure 1:**
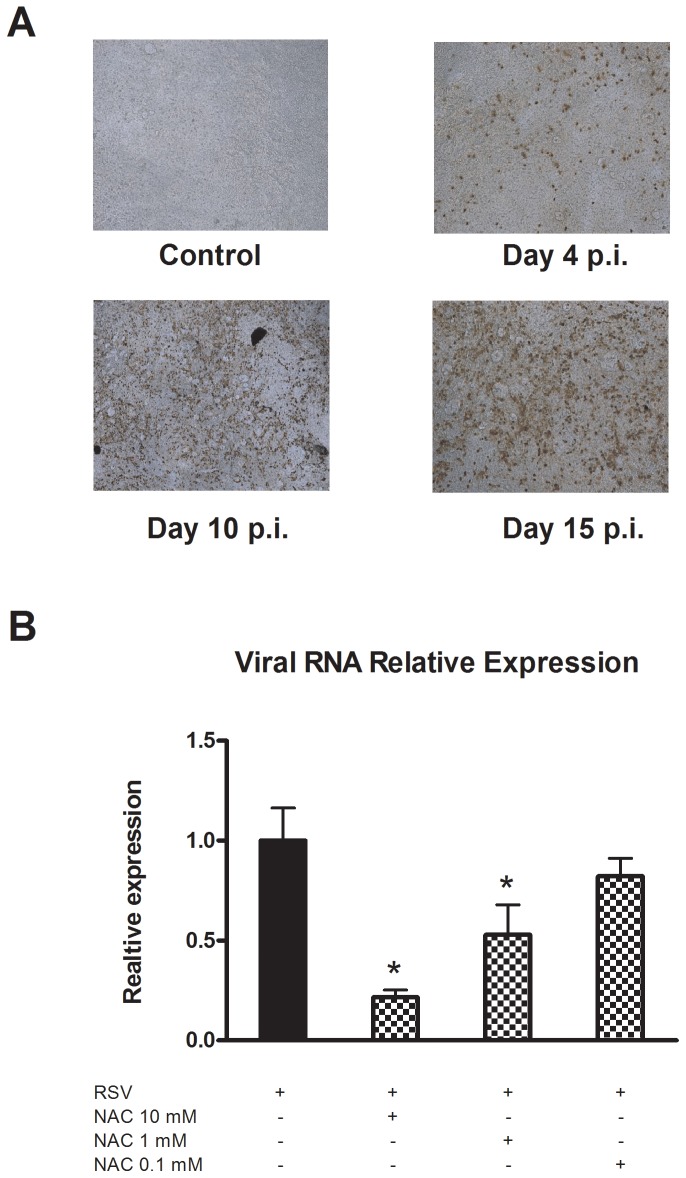
Influence of NAC on RSV replication in differentiated NHBEC cultures. Twenty-one days after ALI differentiation, NHBECs were infected with RSV at 5 PFU per cell in the absence or presence of 0.1, 1 and 10 mM NAC. Virus replication was evaluated by (A) immunochemistry at days 4, 10, and 15 postinfection (p.i.) and by (B) real-time RT-PCR. Experiments were performed in triplicate and six independent infections were used (p = 6, n = 18). *p<0.05, compared to infected cells.

The efficiency of infection was evaluated by immunocytochemistry. NHBECs were grown in an ALI culture system, and 21 days after removal of the apical medium, the cultures were inspected for cilia beat activity and infected with RSV, as described in the Materials and methods. Immunocytochemical analysis of the cultures was done at days 4, 10, and 15 postinfection and compared to mock-infected cultures. Experiments were done in triplicate, and representative results are shown in Fig. 1A. The results indicated that compared to mock-infected cells, the number of RSV-positive cells was significantly high at 4 days after infection and reached its maximum at day 15 postinfection.

The effect of NAC pretreatment on virus replication was determined by real-time RT-PCR. Differentiated cultures were treated with 0.1, 1 and 10 mM NAC for 30 min before infection. Fifteen days later, viral RNA content was measured by real time RT-PCR, as described in the Materials and methods. Experiments were performed in triplicate, and cells from six separate donors were used. The results indicated that NAC significantly inhibited viral infection in a dose-dependent fashion (Fig. 1B). The dose of 1 mM reduced infection by 51% compared to control cells, while the maximum dose used in this investigation (10 mM) almost abolished virus infection of the cultures.

### NAC inhibits the RSV-mediated reduction in the number of active ciliated cells in differentiated NHBECs

**Figure 2:**
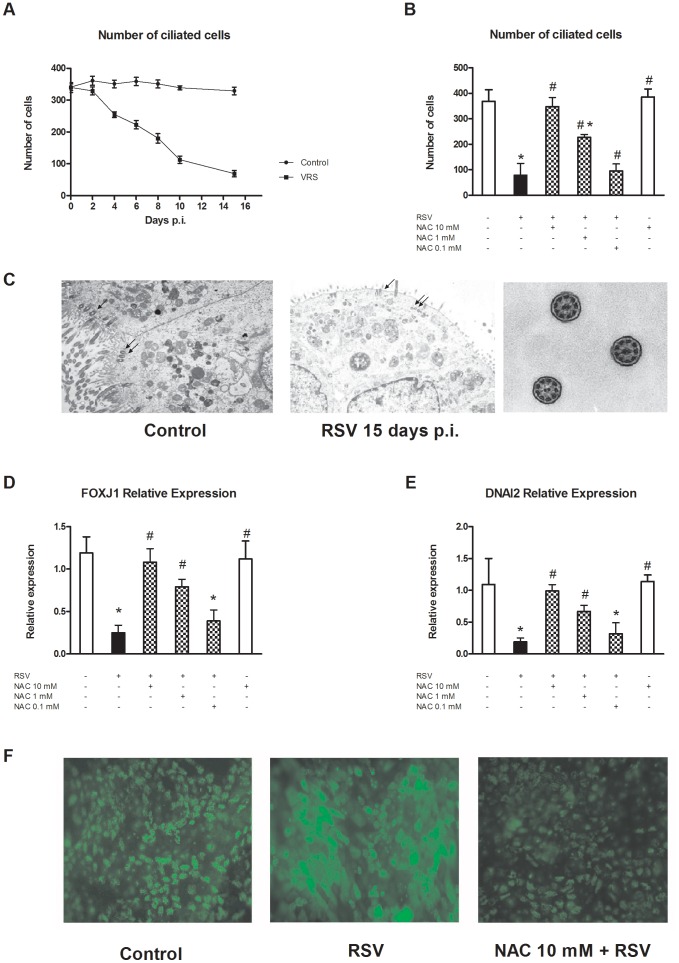
Effect of NAC on ciliary activity and ciliagenesis. Twenty-one days after ALI differentiation, NHBECs were infected with RSV at 5 PFU per cell. Every 48 h after infection, three different videos were recorded using a high-speed video camera. (A) Beating cells were counted. Results are presented as the mean ± SEM of three independent infections. (B) Fifteen days postinfection (p.i.), the effects of NAC were evaluated in mock-infected cultures (white bars) and RSV-infected cells in the absence (black bars) or presence (squared bars) of 0.1, 1 and 10 mM NAC. (C) Ultrastructural alterations in differentiated NHBEC cultures were evaluated by transmission electron microscopy in control (mock-infected cells, left) and RSV-infected (center) at day 15 p.i. Ultrastructures of cilia (right) were also evaluated. Arrows indicate tubular systems. (D) *FOXJ1* and (E) *DNAI2* expression was analyzed by real-time RT-PCR at day 15 p.i. (F) Cultures were also evaluated for β-tubulin expression by immunofluorescence in control (mock-infected cells, left) and RSV-infected cultures in the absence (center) or presence of 10 mM NAC (right). Experiments were performed in triplicate and six independent infections were used (p = 6, n = 18). Data are presented as the mean ± SEM. *p<0.05, compared to mock-infected cells; #*p*<0.05, compared to infected cells.

RSV selectively infects ciliated cells of the airway epithelium, causing a rapid loss of cilia beat activity, which is one of the factors affecting mucus clearance. To analyze this effect in our model of infection, differentiated NHBEC cultures were infected with RSV in the presence or absence of 0.1, 1 and 10 mM NAC. Starting at day 0 and continuing every 48 h after infection, three different high-resolution videos of three independent cultures were recorded. Videos were codified, and the number of cells with ciliary activity was evaluated by counting in a double-blind manner. The results are summarized in Fig. 2A. RSV induced a dramatic reduction of the number of beating cells, reaching statistical significance at day 4 postinfection and its maximum 8 days later (79.03%, compared to mock-infected cells). At this time point, the effect of NAC was evaluated. Results are summarized in Fig. 2B and demonstrate that NAC inhibited the effect of RSV with regard to the number of beating cells in a dose-dependent fashion, reestablishing their numbers to the level of mock-infected cells. The frequency of beating cells was also assessed to determine if, as a consequence of RSV infection, a decrease occurred in the frequency of beating cells. Results showed no difference between the control (12.9±0.79 Hz) and infected (12.49±0.62 Hz) cultures.

### Electron microscopic findings

To elucidate if the loss of beating cells in the cultures infected with RSV was due to a loss of cilia in ciliated cells or to inhibition of ciliary beat movement, ultrastructural studies were performed. Fifteen days after RSV infection, cells were trypsinized and studied under an electron microscope. Results are summarized in Fig. 2C and reveal several abnormalities in infected cells compared to uninfected cultures. In the apical luminal cytoplasm of infected cultures, nondirectional tubulus systems, similar to formations in axonemes, were found (Fig. 2C, arrows). Some of these systems were arranged parallel to the surface; these atypical axonemes were not present in mock-infected cultures. The ultrastructure of cilia were apparently normal in both experimental groups, although the number of cilia in RSV-infected cells was substantially lower than in control cultures, which is in concordance with the number of beating cells. Before trypsinization, and after electron microscopy studies, cells were counted, and no significant differences between control (248×10^3^ cells/insert on average) and infected (236×10^3^ cells/insert on average) cultures were found, which indicated that RSV did not induce cell death in this model, at least at this time point.

### NAC restores the reduction in the expression levels of DNAI2, FOXJ1, and β-tubulin induced by RSV

Results obtained after analysis of the recorded videos and electron microscopy studies pointed to interference of virus infection and ciliagenesis in epithelial cells. To explore this, we decided to analyze the expression of two important genes involved in ciliogenesis, the transcriptional factor *FOXJ1* and the axonemal component *DNAI2*. Our results are presented in Fig. 2D and E, respectively, and demonstrate that 15 days after RSV infection of differentiated cultures, a significant inhibition of the expression of both genes was observed (75% and 81%, respectively). NAC pretreatment of cultures totally abolished this reduction in a dose-dependent manner.

To determine if the downregulation of *FOXJ1* and *DNAI2* correlated with a decrease in ciliated cells, immunofluorescence studies of β-tubulin were performed. The results obtained are presented in Fig. 2F. A strong decrease in β-tubulin-positive cells was observed at day 15 postinfection compared to mock-infected cells. Pretreatment of cultures with 10 mM NAC strongly ameliorated this effect.

### NAC inhibits MUC5AC, GOB5, and IL-13 upregulation in NHBECs infected with RSV: effects on goblet cell metaplasia

**Figure 3:**
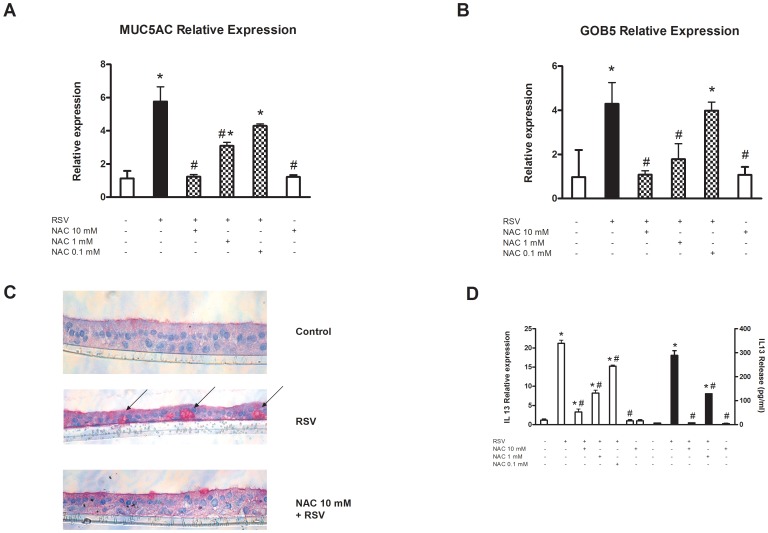
NAC inhibits MUC5AC, Gob5, and IL-13 upregulation and restores the normal structure of epithelium in RSV-infected NHBEC cultures. Twenty-one days after ALI differentiation, NHBECs were infected with RSV at 5 PFU per cell. Total RNA was extracted and analyzed by real-time RT-PCR for (A) *MUC5AC* and (B) *Gob5* expression in mock-infected cultures (white bars) and RSV-infected cells in the absence (black bars) or presence (squared bars) of 0.1, 1 and 10 mM NAC. (C) Histological properties of cultures were evaluated by PAS staining in control (mock-infected cells, upper panel) and infected cultures in the absence (middle panel) or presence (lower panel) of 10 mM NAC. (D) IL-13 expression and release were evaluated by real-time RT-PCR (left, white bars) and Luminex (right, black bars), respectively, in mock-infected cultures and RSV-infected cells in the absence or presence of 0.1, 1 and 10 mM NAC. Protein release was evaluated in culture supernatants. Experiments were performed in triplicate and 6 independent infections were used (p = 6, n = 18). Data are presented as the mean ± SEM. *p<0.05, compared to mock-infected cells; #*p*<0.05, compared to infected cells.

Mucin hypersecretion is another important component that determines mucus clearance by respiratory epithelium. One of the effects observed after RSV infection of epithelial cells was an increase in the expression of mucin *MUC5AC*. To investigate the effect of NAC in this model, differentiated NHBECs were infected with RSV in the absence or presence of 0.1, 1 and 10 mM NAC. Fifteen days after infection, total RNA was extracted from cells, and mucin *MUC5AC* mRNA levels were analyzed by real-time RT-PCR. Our results indicated that after virus infection, a strong induction of the expression of this gene occurred (5.76-fold compared to mock-infected cultures; Fig. 3A). NAC inhibited this upregulation in a dose-dependent manner.

Gob5 is a Ca^2+^-dependent chloride channel that regulates *MUC5AC* expression. Real-time RT-PCR analysis of the expression of this gene revealed a significant increase in its expression (4.29-fold compared to mock-infected cells) that was inhibited by NAC in a dose-dependent manner (Fig. 3B).

To determine if this increase in *MUC5AC* expression was due to an increase in the number of goblet cells, PAS staining was done. NHBEC cultures were infected with human RSV for 15 days in the presence or absence of 1 mM NAC. The results are presented in Fig. 3C. After RSV infection, a decrease occurred in the thickness of the cultures compared to mock-infected cells, as well as an increase in the number of PAS-positive cells (arrows indicated). NAC restored the thickness of the epithelium and the number of goblet cells to normal levels.

Due to the involvement of IL-13 in goblet cell metaplasia, both the expression and release levels of this cytokine were measured in RSV-infected cultures. The results are summarized in Fig. 3D and indicate a significant induction of expression (21.29-fold compared to control cells) and release (45.80-fold compared to mock-infected cells) of IL-13, which was inhibited by NAC pretreatment in a dose-dependent fashion. The dose of 10 mM completely inhibited the induction of this mediator at both the gene and protein expression levels.

### Oxidant events after human RSV infection of NHBECs: effects of NAC

**Figure 4:**
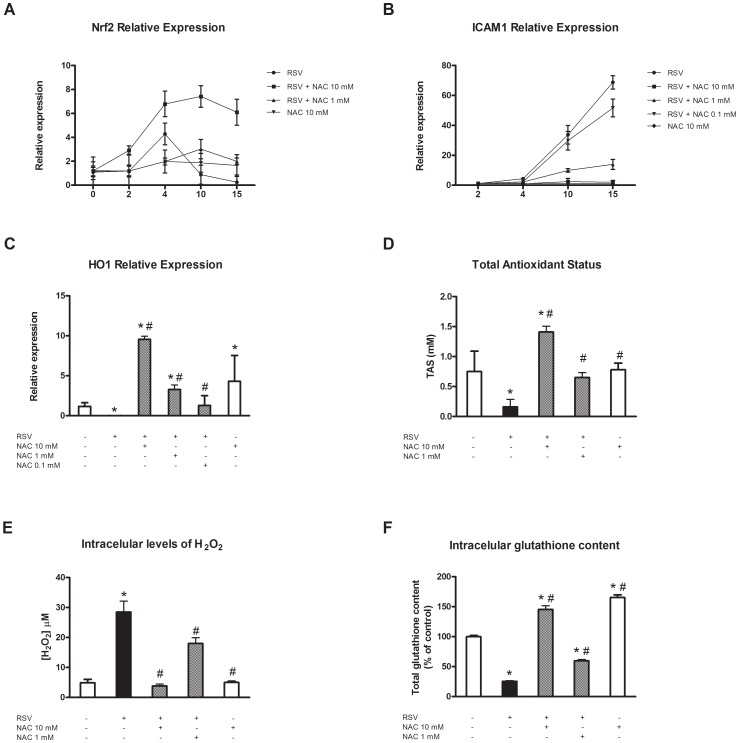
Antioxidant effects of NAC. Twenty-one days after ALI differentiation, NHBEC cultures were infected with RSV at 5 PFU per cell in the absence or presence of 1–10 mM NAC. Total RNA was extracted and analyzed by real-time RT-PCR for (A) *Nrf2* expression, (B) *ICAM1* expression at days 2, 4, 10, and 15 postinfection (p.i.), and (C) *HO1* expression at day 15 p.i. (D) Total antioxidant status, (E) intracellular H_2_O_2_ levels and (F) intracellular glutathione content were analyzed at day 15 p.i. Experiments were performed in triplicate and six independent infections were used (p = 6, n = 18). Data are presented as the mean ± SEM. Experiments were done in triplicate. *p<0.05, compared to mock-infected cells; #*p*<0.05, compared to infected cells. The English in this document has been checked by at least two professional editors, both native speakers of English. For a certificate, please see: http://www.textcheck.com/certificate/QCbgL4.

ROI-mediated effects are clearly involved in the pathogenesis of RSV infection in epithelial cells [16]. In animal models, the early antioxidant response to viral infection is mediated by Nrf2, which controls the expression of several antioxidant genes, including *HO1*. For this reason, we decided to analyze the expression of this protein in our model of infection. Differentiated NHBECs were infected with RSV in the absence or presence of 1 or 10 mM NAC. *Nrf2* expression levels were measured by real-time RT-PCR. The results obtained indicated that after RSV infection, there was a rapid induction of this gene by day 4 postinfection followed by a decrease in mRNA levels to below baseline (0.25-fold compared to control cells at day 15 postinfection; Fig. 4A). This effect was attenuated by 1 mM NAC, but in cultures pretreated with 10 mM NAC, the induction of *Nrf2* was maintained until the end of the experiment. This profile was completely opposite to that found for *ICAM1* expression, as is presented in Fig. 4B. As the levels of *Nrf2* decreased, the expression levels of *ICAM1* increased.

One of the most important known antioxidant genes is *HO1*. Data shown here indicated that the relative expression of this protein was significantly inhibited in RSV-infected cultures at day 15 postinfection (0.15-fold compared to mock-infected cultures; Fig. 4C) and that NAC pretreatment not only restored the levels of *HO1*, but it also induced the its expression to much higher levels than those observed in control cells (9.56 and 3.29-fold with 10 and 1 mM NAC, respectively; Fig. 4C).

TAS determines the response capacity of a biological system to oxidative-mediated events. This status represents the balance between oxidant and antioxidant molecules and can be measured using different approaches. We next decided to analyze whether an alteration of this balance occurred as a consequence of RSV infection of NHBEC cultures. Differentiated NHBECs were infected with RSV in the absence or presence of 1 or 10 mM NAC. Fifteen days later, the antioxidant status of cultures was determined using a TAS antioxidant assay kit. The results obtained are summarized in Fig. 4D and indicate that after virus infection, a decrease occurred in the antioxidant status of the cultures (21% of that of control), which was completely reversed by treatment with 1 mM NAC. Pretreatment with 10 mM NAC induced an increase in TAS of 141% compared to mock-infected cells.

Finally we decide to explore the effects of NAC on intracellular H_2_O_2_ and glutathione content. Result obtained are shown in figure 4 (panel E and F respectively). After RSV infection an increase of intracellular levels of H_2_O_2_ (5.97-fod compared to control cultures) and a decrease of total glutathione levels (25.36% of control) were observed. NAC 10 mM completely restored intracellular H_2_O_2_ to control levels and increased glutathione levels above that of mock-infected cultures.

## Discussion

In this study, we used an *in vitro* model of RSV infection carried out in differentiated cultures of NHBECs to analyze the effects of NAC on the alterations induced by RSV on ciliagenesis and mucin production, the two factors determining mucus clearance. We analyzed the frequency and number of beating cells, the ultrastructural alterations, the expression of two important genes involved in ciliagenesis, and the expression of one of the components of cilia, the β-tubulin protein. In addition, we explored MUC5AC, GOB5, and IL-13 as indicators of metaplasia. Finally, we evaluated the effects of NAC for the modulating oxidative-dependent alterations induced after RSV infection, including *Nrf2* and *HO1* expression, TAS modulation, intracellular H_2_O_2_ levels and intracellular glutathione content.

COPD is the fourth leading cause of death worldwide, accounting for more than 2.5 million deaths every year [20]. Persistent RSV infections are one of the factors associated with COPD exacerbations, which are characterized, among other factors, by an increase in sputum volume [7], [21]. Two principal factors determine mucus clearance in airways: the ciliary beating of ciliated epithelial cells and mucin release [8].

The benefits of NAC inhibiting RSV infection are well characterized in A549 cells [22]. Recently, its capacity to inhibit mucin release in RSV-infected A549 cells was reported [16], but nothing is known about the direct effects of NAC treatment on ciliary activity, ciliagenesis, or metaplasia of respiratory epithelia. Differentiated epithelial cells are one of the accepted models for the study of RSV infection [9]. In this system, RSV specifically infects ciliated cells via the apical membrane [23]. Therefore, these cells constitute a valid model for the study of mucus clearance.

Data presented here demonstrate that NAC significantly inhibited RSV infection of differentiated epithelial cells. This effect may be mediated, at least in part, by ICAM1 and reactive oxygen species (ROS) generation, as discussed later. To our knowledge, this is the first report demonstrating this effect of NAC.

In preliminary experiments the number of beating cells and the determination of viral infection was monitored for 21 days. We found that the decrease in the number of beating cells becomes maximal at day 15 postinfection. This was in parallel with the number of infected cells which reach maximal at day 15 postinfection and remain stable until day 21 (data not shown). This is the reason because we have selected this time point in this study. We have used 5 pfu/cell because with this concentration we obtained maximal infection (data not shown) and is in line with other authors [2], [4]. After RSV infection of differentiated cultures, a rapid decrease occurred in the number of beating cells, which was accompanied by ultrastructural alterations of basal axonemal bodies and a decrease in the number of cilia and β-tubulin-positive cells. These alterations are consistent with those described by others [24]. We also analyzed the frequency of beating cells. Other investigators have reported a rapid decrease in ciliary beat frequency after RSV infection, such that only 2 h after infection, the ciliary beat frequency decreased to 0 Hz in infected cells [24]. We found no differences in the frequency of beating cells in RSV-infected cultures, probably because our system only allowed us to measure the frequency of cells that were beating, and these cells likely had not yet been infected by the virus. In this study, we also analyzed the expression of two important genes related to ciliagenesis, *FOXJ1* and *DNAI2*. *FOXJ1* is a member of the forkhead/winged-helix family of transcription factors that regulates the expression of several genes implicated in motile cilia structure, including those encoding axonemal dyneins [25], calpastatin [26], and components of the central complex like WDR16 or centrin2 [27]. *DNAI2* is implicated to be involved in the outer dynein arm assembly [28]. The gene expression results of this study correlated with those observed for the number of ciliated cells; they showed that the expression levels of both genes may be indicative of the number of ciliated cells in the airway epithelium and may be considered as markers of metaplasia in airways. NAC pretreatment of cultures inhibited, in a dose-dependent fashion, all of these changes, restoring the normal ciliary activity of cultures.

The viscosity of mucus is determined by different factors, including mucin release, of which MUC5AC is the most prominent, in human airways [12]. MUC5AC expression is controlled by the Ca^2+^-dependent chloride channel, GOB5 [29]. We observed a significant upregulation of the expression of both genes in our model, which was inhibited in a dose-dependent manner by NAC. Histological studies indicated that this increase in *MUC5AC* expression was accompanied by changes in the thickness and density of goblet cells, which were restored by NAC pretreatment of cultures. These observations were in accordance with the induction of IL-13 observed after RSV infection, which has been reported as critical for epithelium metaplasia and with the cilia loss in human airway epithelium [30], [26]. Animal models studies demonstrate that the induction if IL-13 release is one of the key regulators of the inflammatory response against RSV infection [31], [32], [33] which is in line with data presented here. Because IL-13 expression by airway epithelial cells is controversial, we have used two different approaches to measure it. On the one hand we have study the expression of IL-13 gene using Real-Time RT-PCR, and on the other hand we have measured the protein levels in culture supernatants using a R&D designed luminex assay. Both systems have been used to study the expression of this cytokine in epithelial cells [34], [35], [36].

All the effects of NAC can be explained by its capacity to inhibit viral infection, as supported by the results presented here. The cell surface adhesion molecule ICAM1 is essential for RSV infection of epithelial cells. This protein binds to F glycoprotein and facilitates virus entry into the host cell [37]. Increases in ICAM expression after RSV infection of bronchial epithelial cells has been previously reported and determines the infectivity of RSV [38], [39]. The expression of ICAM1 is controlled by ROS-mediated events [40]. Our findings are in line with these observations, as after RSV infection we detected a strong induction of ICAM1 expression in differentiated NHBEC cultures, which was inhibited by NAC. The modulation of oxidative stress observed here can explain the inhibition of ICAM1 overexpression and the subsequent inhibition of viral infection of differentiated NHBECs.

One known factor involved in the control of antioxidant genes, like HO-1 or glutathione peroxidase (GPx), is the nuclear factor Nrf2 [41]. In agreement with observations made in animal models, we observed a rapid increase in *Nrf2* expression followed by a decrease to levels lower than those observed in non-infected cells [42]. Probably this pattern indicate the physiological early response of cultures to virus infection and was dramatically changed by NAC, which induced HO-1 expression and restored the antioxidant status of cells. Cho et al. analyzed the upregulation of HO-1 gene in an animal model of RSV infection and found, in parallel to Nrf2 pattern, a rapid increase in the expression of this antioxidant protein, followed by a decrease until levels lower than those found in the control animals [42]. We have analyzed the expression of HO-1 at day 15 postinfection and our results indicate, in concordance to these observed by Cho et al. lower levels of expression of this gene compared to mock-infected cells. NAC induces a significantly increase in the levels of HO-1, which can explain the effects observed at the total antioxidant status and in the intracellular H_2_O_2_ levels of treated cultures compared to untreated. NAC is a known precursor of glutathione, the most important intracellular antioxidant molecule [43], [44]. Data supported here indicate that after RSV infection there is a significantly decrease of glutathione levels which are increased in NAC treated cultures. This increase explain the restore of intracellular H_2_O_2_ levels and the inhibition of ICAM expression with subsequent reduction of RSV capacity to infect cultures cells.

In summary, we conclude that NAC inhibited RSV infection in differentiated NHBEC cultures, restoring normal ciliary activity and inhibiting mucin release. These results support the beneficial effects of NAC treatment for the management of respiratory diseases, including COPD, in which 64% of exacerbations are associated with respiratory virus infection.

## Materials and Methods

### Cell model and experimental groups

Human lung tissue was obtained from patients who had undergone surgery for lung carcinoma, as previously outlined [45]. Experiments were approved by the local ethics committee and informed consent was obtained. At the time of operation, lung function was within normal limits by spirometry. None of the patients were being chronically treated with theophylline, β-adrenoceptor agonists, corticosteroids, or anticholinergic drugs. Bronchia were carefully dissected free from adjoining connective tissue and lung parenchyma. Human bronchial epithelial cells were cultured and differentiated in 24-well Transwell inserts (0.3 cm^2^; Corning Costar, High Wycombe, UK) under ALI conditions, as previously described [46]. In brief, a multilayered bronchial epithelium was obtained by seeding cells (8.25×10^4^ cell per insert) onto polyester inserts (Millipore, Billerica, MA). Cells were submerged in differentiation medium [50% Dulbecco's modified Eagle's medium (DMEM) in basal epithelial growth media (BEGM); Clonetics, Wokingham, UK] for the first 7 days. Cells were then cultured for an additional 21 days with the apical surface exposed to air, and ciliary activity was inspected every day until the maximal density of ciliated cells (200–250 ciliated cells per field) was reached. Cultures were then washed three times with fresh differentiation culture medium and infected with 100 µL of the same media containing 2×10^6^ plaque forming units (PFU) RSV per insert. Cultures were incubated for 2 h at 37°C and washed once with 500 µL differentiation media. In this study, we used cultures from six different donors. All experiments were done by triplicates (p = 6, n = 18).

Infected cultures were maintained until day 15 postinfection. Culture medium was replaced every 48 h. Cilia activity, culture supernatants, and cells were collected at the indicated time points. In this study, the following experimental groups were included: control (untreated and mock-infected cells), infected (cells infected with RSV), infected and treated (cells infected with RSV in the presence of 0.1, 1 and 10 mM NAC; Sigma-Aldrich, St. Louis, MO), and only treated (mock-infected cells treated with 0.1, 1 and 10 mM NAC). NAC was dissolved in distilled water as indicated by the manufacturer, added to the culture medium 1 h before infection and maintained until the end of the experiment. Cell toxicity of the three different concentrations of NAC used in this study was analyzed using the presto blue cell viability reagent (Invitrogen Ltd., Paisley, UK) following manufacturer's instructions. No effect of any of the concentrations tested was observed.

### Preparation of the virus

RSV (long Strain, ATCC VR-26) obtained from the American Type Culture Collection (ATCC, Rockville, MD) was propagated in Hep-2 cells in DMEM (Invitrogen Ltd., Paisley, UK) with 2% fetal calf serum (DMEM-2; Invitrogen Ltd.), as previously described [47]. Viruses were purified from clarified culture supernatants by polyethylene glycol precipitation and centrifugation in a 30–45–60% discontinuous sucrose gradient in TNE buffer [48], [49]. Virus titters were determined by plaque assay in Hep-2 cells layered with 0.5% low melting- point agarose (Conda Laboratories, Madrid, Spain). After 5 days, cells were fixed with 4% formaldehyde (PanreacQuimica, Barcelona, Spain) in phosphate-buffered saline (PBS), followed by methanol, and incubated with a mixture of monoclonal antibodies against the two major glycoproteins of the virus (2F, 47F, 56F, 021/1G, 021/2G; Sigma-Aldrich) [47], [49]; plaques were visualized using an anti-mouse IgG horseradish peroxidise-linked whole antibody (Amersham Pharmacia Biotech Europe GmbH, Freiburg, Germany) and 3-amino-9-ethylcarbazole (AEC; Sigma-Aldrich). Virus inactivation was achieved by irradiation with ultraviolet light for 90 min and confirmed by plaque assay.

### Determination of viral infection

In this study, we used two methods to determine viral infection of differentiated NHBEC cultures. The first method was immunocytochemical detection of viral glycoproteins (2F, 47F, 56F, 021/1G, 021/2G), as described above, and the second method was semiquantitative real-time reverse transcription polymerase chain reaction (RT-PCR) with primers designed to amplify nucleoprotein RNA (forward primer, 5′-CATGATTCTCCTGATTGTGGGATGA-3′; reverse primer, 5′-TCACGGCTGTAAGACCAGATCTAT-3′; probe, 5′-CCCCTGCTGCCAATTT-3′) [50]. RNA extraction, cDNA synthesis, and real-time PCR were done as described below.

### Ciliary beat and ultrastructure analysis

Ciliary motility activity and frequency were evaluated as described previously [51]. Every 48 h after infection, five videos of each insert were recorded using a high-speed video digital camera. The number of cells with ciliary activity was measured by counting under double-blind conditions to minimize experimental errors due to the observer.

Fifteen days postinfection, cells were trypsinized and processed for transmission electron microscopy, as outlined previously [51]. Briefly, cells were fixed in 0.03 M phosphate buffer containing 2.5% glutaraldehyde for 1 h. Then, the cells were rinsed in the same buffer for 30 min and fixed in 1% osmium tetroxide for 1 h. The most representative areas were selected and examined with a transmission electron microscope.

### Immunofluorescence analysis of β-tubulin

For β-tubulin immunofluorescence analysis, nitrocellulose membranes (Amersham Pharmacia Biotech Europe GmbH) were ethanol-fixed and incubated with a mouse monoclonal antibody against β-tubulin (Sigma-Aldrich) and a secondary rhodamine anti-mouse fluorescein isothiocyanate (FITC)-conjugated antibody (Sigma-Aldrich). Images were acquired using a fluorescence microscope. Five different images of each insert were analyzed.

### Goblet cell analysis

To estimate the number of goblet cells in the differentiated NHBEC cultures, periodic acid-Schiff (PAS) staining was done. Nitrocellulose membranes were removed from the inserts, fixed, and paraffin-embedded, as previously outlined [45]. Inserts were cut in 5-µM sections and stained using the PAS staining system (Sigma-Aldrich). Finally, sections were mounted with DPX (Sigma-Aldrich) and analyzed by microscopy.

### Determination of MUC5AC, ICAM1, Gob-5, IL-13, Nrf2, and HO1 mRNA expression

Mucin *MUC5AC* mRNA transcripts were measured by real-time RT-PCR, as previously reported [45]. Total RNA was isolated with TRIzol reagent (Invitrogen Ltd.) following the manufacturer's instructions. Integrity was measured with a 2100 Bioanalyzer (Agilent Technologies Inc., Santa Clara, CA). Only extractions with an integrity ratio (28S/18S) near 2.0 were considered. Two-hundred nanograms of total RNA were retro-transcribed into cDNA using TaqMan RT reagents (N808-0234; Applied Biosystems, Foster City, CA), as indicated by the manufacturer. *GAPDH* was used as an endogenous control. Primers and probes for both *MUC5AC* and *GAPDH* (Sigma-Aldrich) were designed using Primer Express software, as previously reported [45]. For the rest of the genes included in this study, Assays-on-Demand™ (Applied Biosystems) was used. The ΔΔC_t_ method was used to obtain semi-comparative data.

### Determination of IL-13 protein in supernatants

At the indicated time points, cultures were washed once with 100 µL sterile PBS, and 65 µL fresh culture medium was added to the surface. After 1 h of incubation, supernatants were collected and analyzed for IL-13 release using a multiplex cytometer-based method (Luminex DX100; Luminex Corp., Austin, TX). Panels and specific bead-conjugated antibodies were purchased from Millipore, and calculations were carried out following the manufacturer's instructions. Ultrasensitive panels were used for these experiments.

### Determination of Total Antioxidant Status (TAS)

TAS was determined using the Total Antioxidant Assay kit (Cayman Chemical Company, Ann Arbor, MI) following the manufacturer's instructions. This assay relies on the ability of antioxidants in the sample to inhibit the oxidation of ABTS (2,2′-azino-di-[3-ethylbenzthiazoline sulfonate]) to ABTS+ by metmyoglobin. The capacity of the antioxidants in the sample to prevent ABTS oxidation is compared with that of Trolox, a water-soluble tocopherol analog. Results are expressed as molar Trolox equivalents.

### Determination of intracelular levels of H_2_O_2_

Intracellular levels of H_2_O_2_ were determined using the Amplex Red Reagent as previously described [16]. In briefly, cultures were lysed in 100 µM Amplex red solution supplemented with 2 U/ml HRP and 200 mU/ml superoxide dismutase (OXIS International Inc., Beverly Hills, CA, US) and incubated in the dark for 30 min. Fluorescence was measured in a plate reader at excitation/emission wavelengths λ_EX_ = 540 nm/λ_EM_ = 590 nm, respectively.

### Determination of intracellular glutathione content

Cultured cells were lysed in chilled 1% SDS lysis buffer. Total glutathione levels were determined by measuring the conversion of 55′-dithiobis(2-nitrobenzoic acid) in the presence of GSH to 2-nitro-5-thiobenzoic acid, using the total glutathione quantification kit (Dojindo, Rockville, MD, USA). Sample absorbance was read at 415 nm and data were normalized to total protein, as determined by a standard protein concentration assay (Biorad, Hercules, CA, USA).

### Analysis of results

Data are presented as the mean ± SEM. Statistical analysis of the results was carried out by analysis of variance (ANOVA) followed by Tukey's multiple comparison test or *t*-test, as appropriate (GraphPad Software Inc., San Diego, CA). Significance was accepted when *p*<0.05.
